# Gasless submental-transoral combined approach endoscopic thyroidectomy: a new surgical technique

**DOI:** 10.3389/fonc.2023.1115927

**Published:** 2023-05-31

**Authors:** Jinxi Jiang, Gaofei He, Junjie Chu, Jianbo Li, Xiaoxiao Lu, Xianfeng Jiang, Lei Xie, Li Gao, Deguang Zhang

**Affiliations:** Department of Head and Neck Surgery, Sir Run Run Shaw Hospital, Medical School of Zhejiang University, Hangzhou, Zhejiang, China

**Keywords:** thyroid cancer (TC), endoscopic thyroidectomy (ET), gasless, submental, transoral

## Abstract

**Background:**

The development of transoral endoscopic vestibular approach thyroidectomy (TOETVA) has been limited by inherent defects, such as mental nerve injury and carbon dioxide (CO_2_)-related complications. Herein, we proposed a new technique without CO_2_ called gasless submental-transoral combined approach endoscopic thyroidectomy (STET) to solve the problems in TOETVA.

**Methods:**

We reviewed 75 patients who successfully underwent gasless STET using novel instruments at our institution from November 2020 to November 2021. A main incision of approximately 2 cm was made in the natural submental crease line and then combined with two vestibule incisions to complete the procedure. Demographic data, surgical technique and perioperative outcomes were retrospectively recorded.

**Results:**

Thirteen male and sixty-two female patients with a mean age of 34.0 ± 8.1 years were enrolled in this study. Sixty-eight patients had papillary thyroid carcinomas and seven had benign nodules. We successfully performed all gasless STET without conversion to open surgery. The average postoperative hospital stay was 4.2 ± 1.8 days. One transient recurrent laryngeal nerve injury and two transient hypoparathyroidisms were observed. Three patients complained of slight lower lip numbness on the first postoperative day. One case of lymphatic fistula, subcutaneous effusion, and incision swelling occurred each, all of which were conservatively cured. One patient developed a recurrence six months after surgery.

**Conclusions:**

Gasless STET using our own designed suspension system is technically safe and feasible with reasonable operative and oncologic results.

## Introduction

The growing focus on cosmetic results and progress in medical technology has accelerated the development of minimally invasive procedures. Compared with traditional open thyroid surgery, endoscopic thyroidectomies *via* various approaches have gained universal favor since they were employed to bypass the anterior cervical incision, including subclavian (1), axillary (2–4), breast (5), oral (6), and hybrid of oral and submental approaches (7). Nonetheless, for these conventional endoscopic procedures, carbon dioxide (CO_2_) insufflation for maintaining the working space remains an inherent limitation, since it may cause CO_2_ embolism, pneumothorax, and subcutaneous emphysema (8, 9). CO_2_ embolism can be fatal although it is a rare event (8, 9). To overcome this critical problem, an increasing number of surgeons have attempted endoscopic thyroidectomies without any gas insufflation *via* various remote access (10–14). However, these remote-access techniques do not comply with truly minimal invasiveness because an extensive dissection of subcutaneous tissue is required to reach the target site, except for gasless transoral endoscopic thyroidectomy *via* the vestibular approach (TOETVA). TOETVA emerging as a type of natural orifice transluminal endoscopic surgery (NOTES), has gained popularity over a relatively short time due to a more direct surgical route, shorter dissection distance, less invasion, and bilateral thyroid gland exposure (15, 16). However, some unavoidable weaknesses of TOETVA, including discomfort at the chin and lower lip caused by mental nerve injury and flap dissection, the difficult establishment of operative space, and the limitation of large tumor intact extractions *via* the chin due to anatomic obstacles, cannot be ignored. To overcome the above problems of TOETVA, a novel technique called a hybrid transoral and submental thyroidectomy (TOaST) seemed to have been preliminarily confirmed to simplify the TOETVA procedure and achieve aesthetic results equal to those of TOETVA, but it did not use the gasless insufflation technique (7). Use the gasless insufflation technique.

In this study, we designed an innovative retractor-suspension method to create a surgical space to accomplish gasless submental-transoral combined approach endoscopic thyroidectomy (STET), with the main advantage of preventing mental nerve injury and avoiding CO_2_-related complications. Here, we attempted to present our preliminary experience and discuss the feasibility, and safety of gasless STETs.

## Materials and methods

### Patient selection

We reviewed demographic and clinical data from 75 consecutive patients who underwent gasless STET at the Department of Head and Neck Surgery, Sir Run Run Shaw Hospital, Zhejiang University School of Medicine from November 2020 to November 2021. The eligibility criteria were as follows: (1) papillary thyroid cancer (PTC), which was confirmed by fine-needle aspiration (FNA) preoperatively, up to 3 cm in maximum diameter, and benign nodules up to 6 cm in maximum diameter, estimated by ultrasound; (2) no lymph node metastasis in the lateral neck compartment; (3) no history of neck surgery or radiotherapy; and (4) patients with protruding chins or submental depressions who required an aesthetic incision. The exclusion criteria were: (1) infections of the chins or oral cavity; (2) patients with double chins; (3) patients with scar diathesis; and (4) invasion of the trachea, esophagus, and recurrent laryngeal nerve by primary tumor or metastatic lymph nodes. Approval by the ethical committee and informed consent from all patients were obtained.

### Surgical instruments

**Figure 1:**
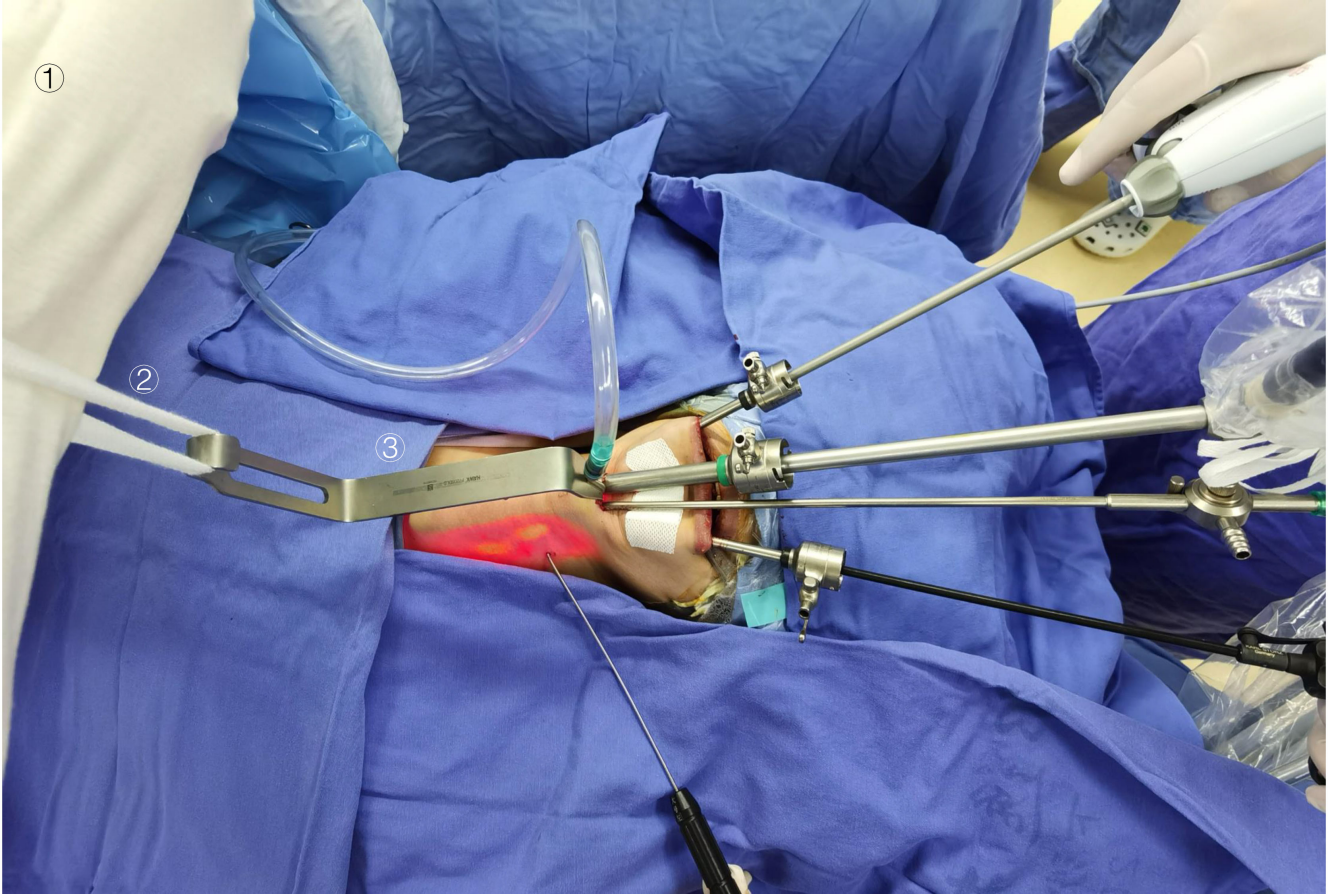
Establishment of the operating space for gasless STET using our own designed suspension system and other main endoscopic instruments. **①** an anesthesia stand, **②** a sterile bandage, **③** a self-developed retractor.

**Figure 2:**
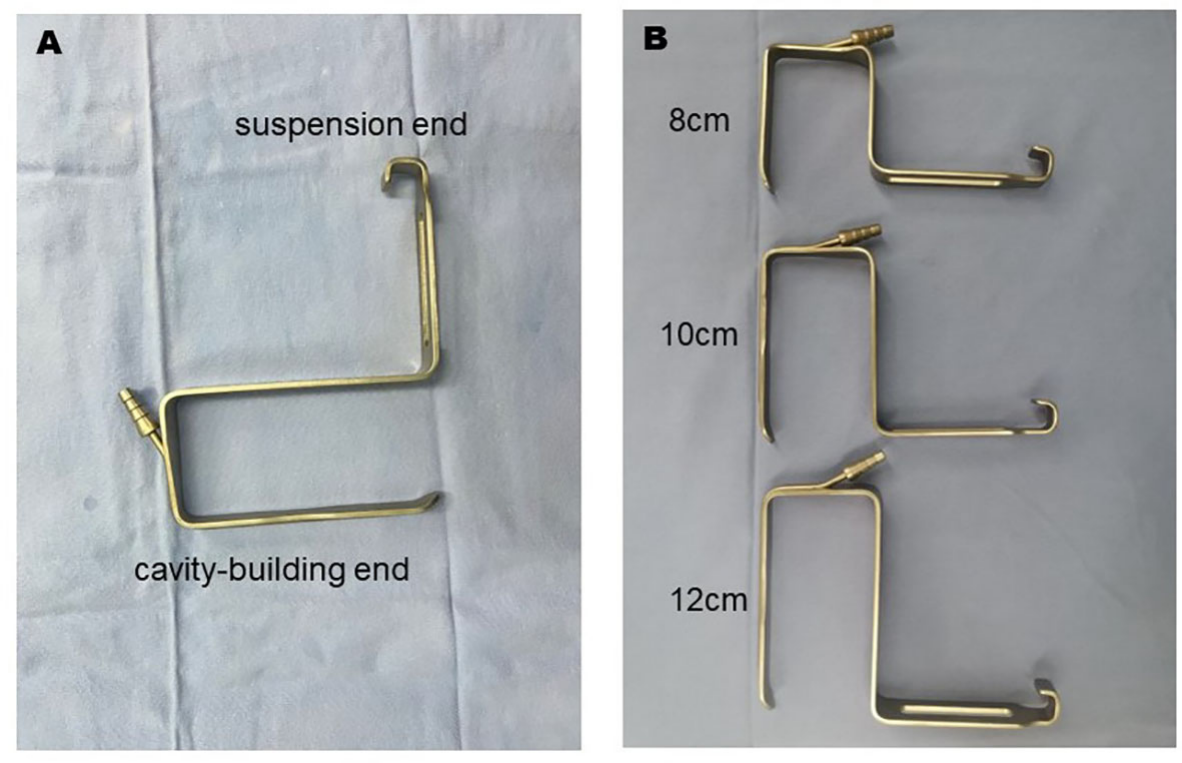
The independently developed retractors with a suction device. **(A)** Details of the new retractor. **(B)** Three retractors of different lengths (8 cm, 10 cm, and 12 cm).

Our self-designed suspension system consists of a retractor, sterile bandage, and anesthetic frame. Each retractor had a suspension end and a cavity-building end. The suspension end is hung on the anesthesia frame by a sterile bandage, and the cavity-building end holds up the neck skin flap to create the surgical space, as shown in **Figure 1**. This novel retractor is particularly equipped with a suction head, which could be directly connected to the suction tube (**Figure 2A**). Such a particular structure facilitates the timely discharge of smoke and maintained a clear surgical field of vision at all times so that the operation could be performed without interruption. What’s more, our suspension retractors include three different lengths (8 cm, 10 cm, 12 cm) to meet all types of neck lengths (**Figure 2B**). In our experience, the whole suspension system avoided the disadvantages of manual retractors, which are easily fatigued, and kept the surgical operating space stable and safe for a long time. The complete set of instruments was very simple to use and allowed the successful completion of the operation without CO_2_ insufflation.

### Surgical technique

For all patients, preoperative laryngoscopy was routinely completed to assess vocal cord motion, and intraoperative nerve monitoring (IONM) was applied to aid in the identification and protection of the laryngeal recurrent nerve.

**Figure 3:**
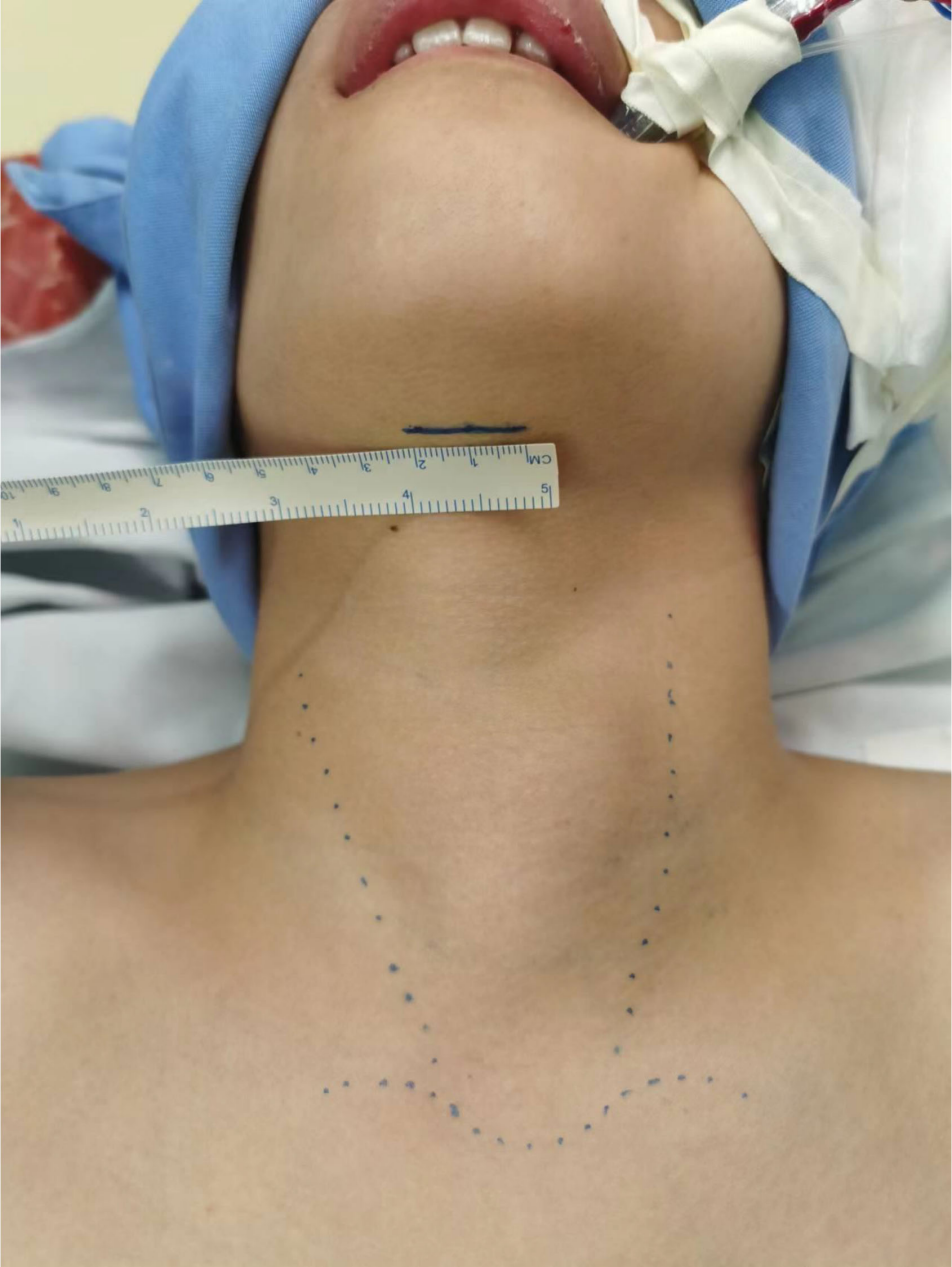
Markers on the body surface before operation.

Before the patient was anesthetized, we needed to mark the body surface locations of the suprasternal notch, bilateral superior clavicular margins, and bilateral anterior sternocleidomastoid margins and mark the location of the 2-cm incision for the observation port, located on the natural submental crease line (**Figure 3**). The anesthesia stand was mounted at the level of the patient’s shoulder in preparation for surgical space establishment before routine disinfection and draping.

The complete surgical procedure consisted of two steps: the first was the creation of the surgical operating space, and the second was thyroid gland resection and central lymph node dissection. First, we made a 2-cm incision at the submental mark as the first incision. Then, we separated down to the deep surface of the platysma and widened the surgical operating space with an electric scalpel as far as possible toward the suprasternal notch under direct vision, which was the first operating space. The other two longitudinal incisions, 5 mm in size, were located lateral to the canine teeth and on the lower lip close to the free edge to avoid mental nerve injury, as in TOETVA (17, 18). Two 5-mm-diameter trocars made for operational ports were inserted into the first operating space through these two incisions to help establish the second working space. Both a 10 mm-diameter high-definition endoscope and a homemade suspension retractor with a continuous suction device were placed in the submental incision. The skin flap was lifted upward with a suspension retractor that was suspended from the anesthesia frame with a sterile bandage. We used an ultrasound scalpel to dissect sharply along the platysma muscle’s deep surface under the endoscopy field to reach the suprasternal fossa and to reach the anterior margin of the sternocleidomastoid muscle on both sides. As the operation progressed, the appropriate length of the suspension retractor was selected according to the position of the operating point. At this point, the second operating space was established.

**Figure 4:**
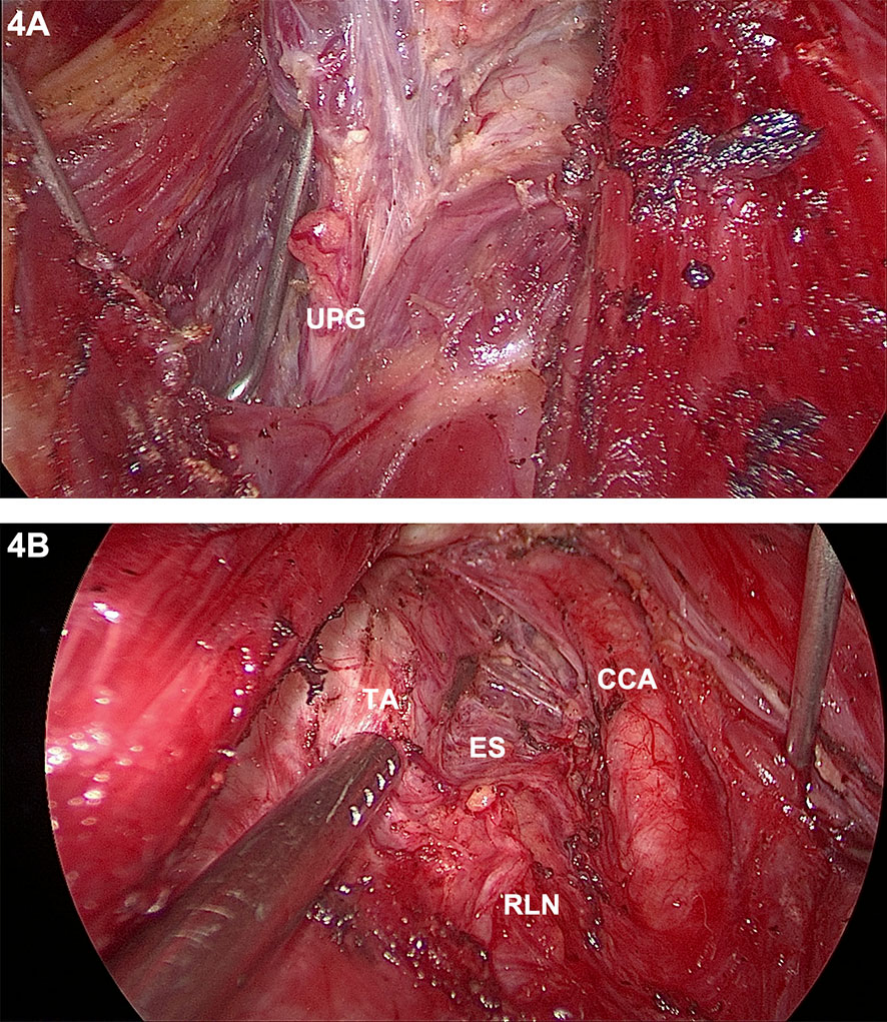
Photographs during gasless STET: Fine dissection of the dorsal thyroid capsule **(A)**; Endoscopic view after completion of central lymph node dissection **(B)**. UPG, upper parathyroid gland; TA, trachea; ES, esophagus; RLN, recurrent laryngeal nerve; CCA, common carotid artery.

**Figure 5:**
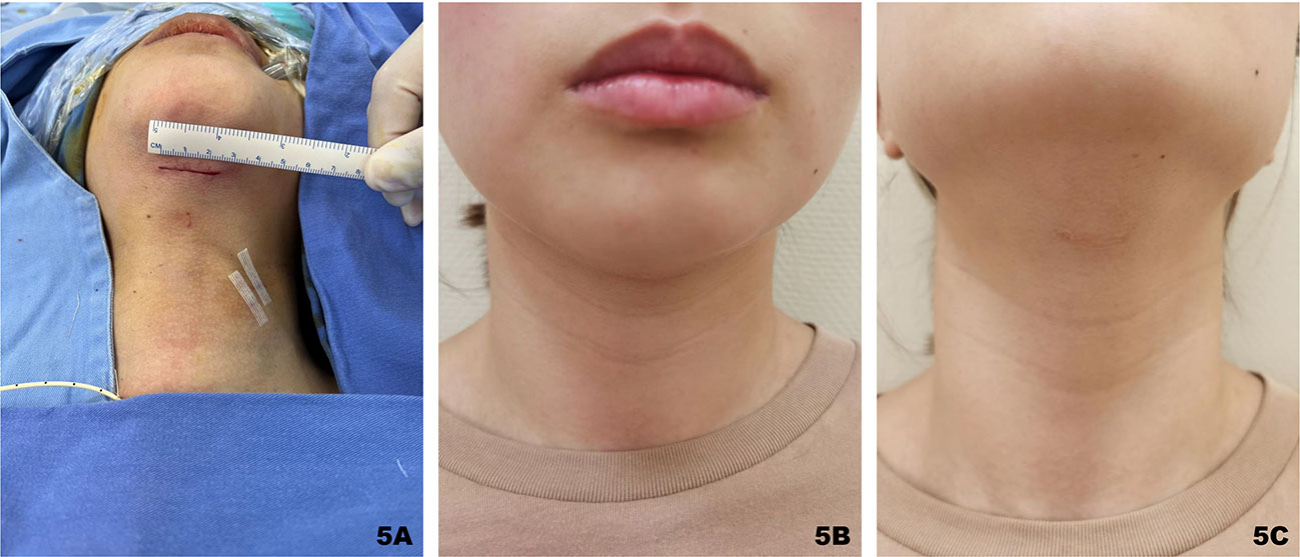
Submental incision shown immediately after surgery **(A)**; One month after the operation, the incision in the horizontal view was hidden under the chin **(B)**; the incision was not obvious even in the head-up position **(C)**.

The procedures of thyroid resection and central lymph node dissection were routinely performed step by step with IONM as in TOETVA (19). After the separation reached the carotid sheath, the strap muscles of each side were retracted separately to the ipsilateral side with the help of the percutaneous thyroid pulling hook to better expose the operative area. As with conventional thyroidectomy, the parathyroid gland and the recurrent laryngeal nerve (RLN) should be carefully dissected and protected using the fine membrane dissection technique during the procedure (**Figure 4A**) After completion of thyroidectomy and central lymph node dissection, the anatomy in the endoscopic view was shown in **Figure 4B**. At the end of the operation, the drainage tube was placed routinely on the opposite side of the neck so that it was not easily bent and at a lower position which was conducive to adequate drainage. What’s more, the drainage tube is thin enough and it does not affect the aesthetic appearance of the healing. The appearance of the skin after suturing the submental and oral incisions was shown in **Figure 5A**.

During the follow-up, an ultrasound assessment of the thyroid bed area and lateral neck lymph nodes was performed every six months after the operation. FNA was further performed if any lymph node of suspicious malignancy was found (20).

## Results

### Demographic data

The demographics and clinical results were listed in detail in **Table 1**. 13 patients were male and 62 patients were female, and the mean age was 34.0 ± 8.1 years (range, 19-58 years). The postoperative pathology diagnosis was PTC in 68 patients and benign nodule in 7 patients. The mean tumor size was 8.2 ± 4.9 mm (range, 2.6 - 30 mm) in PTC cases and 36.3 ± 11.2 mm (range, 21.0 - 52.2 mm) in benign cases. The pathologic tumor stage distribution of the 68 patients with PTC was as follows: 62 pT1 cases (91.2%), 3 pT2 cases (4.4%), and 3 pT3 cases (4.4%). The lymph node stage was pN0 in 36 cases and pN1a in 32 cases.

**Table 1:** Demographic and clinical data of the study population (n=75).

Variables	Value	Range/Percent
**Age (mean ± SD, years)**	34.0 ± 8.1	19-58
Gender		
** Male**	13	17.3%
** Female**	62	82.7%
Tumor size (mean ± SD, mm)		
** Total**	10.8 ± 10.0	2.6-52.2
** PTC**	8.2 ± 4.9	2.6-30
** Benign**	36.3 ± 11.2	21.0-52.2
Pathology		
** PTC**	68	90.7%
** Benign**	7	9.3%
T stage		
** pT1**	62	91.2%
** pT2**	3	4.4%
** pT3**	3	4.4%
N stage		
** pN0**	36	52.9%
** pN1a**	32	47.1%

### Operative details and surgical outcomes

The surgical outcomes of gasless STETs were summarized in **Table 2**. All surgeries were successfully completed by endoscopy without conversion to open surgery. There were 7 lobectomies, 52 lobectomies + central neck dissections (CNDs), 7 total thyroidectomies + unilateral central neck dissections (UCNDs), and 9 total thyroidectomies + bilateral central neck dissections (BCNDs) performed. The average operative time for all STETs was 141.2 ± 31.6 minutes, with a mean operative time of 128.6 ± 25.1 minutes for lobectomy, 141.1 ± 34.8 minutes for lobectomy + CND, 145.0 ± 22.2 minutes for total thyroidectomy +UCND and 148.9 ± 20.6 minutes for total thyroidectomy + BCND. The mean number of central lymph nodes retrieved from PTC patients was 7.2 ± 4.5, with a mean of 1.4 ± 2.4 positive lymph nodes. The mean postoperative hospital stay was 4.2 ± 1.8 (range: 2-17) days.

**Table 2:** Operative details and surgical outcomes of gasless STETs (n=75).

Variables	Value	Range/Percent
Extent of Surgery		
** Lobectomy**	7	9.3%
** Lobectomy + CND**	52	69.3%
** Total thyroidectomy + UCND**	7	9.3%
** Total thyroidectomy + BCND**	9	12.0%
**Conversion to open surgery**	0	0.0%
Operative time (mean ± SD, min)		
** Lobectomy**	128.6 ± 25.1	80-155
** Lobectomy + CND**	141.1 ± 34.8	80-225
** Total thyroidectomy + UCND**	145.0 ± 22.2	120-185
** Total thyroidectomy + BCND**	148.9 ± 20.6	120-185
** Total**	141.2 ± 31.6	80-225
**No. of dissected LNs (mean ± SD, piece)**	7.2 ± 4.5	1-25
**No. of LN metastasis (mean ± SD, piece)**	1.4 ± 2.4	0-11
**Postoperative hospital stay (mean, days)**	4.2 ± 1.8	2-17
**Follow-up time (mean, months)**	13.6 ± 0.346	6-18
Complications		
**Recurrent laryngeal nerve paralysis**		
** Transient**	1	1.3%
** Permanent**	0	0.0%
Hypoparathyroidism		
** Transient**	2	2.7%
** Permanent**	0	0.0%
**Cutaneous paralysis in the lower lip**	3	4.0%
**Hematoma**	0	0.0%
**Subcutaneous fluid**	1	1.3%
**Subcutaneous emphysema**	0	0.0%
**Pneumomediastinum**	0	0.0%
**Chylous fistula**	1	1.3%
**Surgical site infection**	1	1.3%
**Tracheal injury**	0	0.0%
**Esophageal injury**	0	0.0%
**Recurrence**	1	1.3%

CND, central neck dissection; UCND, unilateral central neck dissection; BCND, bilateral central neck dissection; LN, lymph node.

One RLN injury resulting in vocal fold paralysis was observed but recovered within 6 months. Two patients suffered from hypoparathyroidism, but it was transient. Slight sensory loss of the lower lip occurred in 3 patients on the first postoperative day and full recovery after one month. There was 1 case each of lymphatic fistula, subcutaneous effusion, and incision redness and swelling, all of which were conservatively cured. There was no evidence of postoperative bleeding, tracheal or esophageal injury, or other complications. The submental incision, hidden under the chin in a normal standing position, healed well 1 year after the operation without scar formation (**Figures 5B, C**).

The mean follow-up period was 13.6 ± 0.3 months (range 6-18 months). Of the 75 patients who underwent gasless STET, one patient had suspected lateral neck lymph node metastasis by ultrasound examination at 6 months postoperatively and confirmed by FNA. Due to the small size of the metastatic lymph node, follow-up observation was selected.

## Discussion

To meet the demand for cosmetic incisions, the safety, efficacy and feasibility of minimally invasive thyroidectomy *via* various accesses have been evaluated and verified since the first endoscopic thyroid surgery was performed by Hüscher et al. in 1997 (21). Among these remote-access approaches for endoscopic thyroidectomy, the TOETVA and TOaST passing through the natural space are preferred because of the additional trauma to the patient associated with other approaches. However, an endoscopic thyroidectomy regardless of the approach requires air insufflation to retain the operating space. In the process of establishing a workspace with blunt instruments, some small vessels would inevitably be damaged. As such, there is a higher likelihood of CO_2_ gas entering the circulatory system with life-threatening consequences, including subcutaneous emphysema, pneumothorax, pneumomediastinum, and CO_2_ embolism (8, 9). Video-assisted neck surgery (VANS) using a subclavian approach was proposed as early as 1998, which did not need air insufflation and did not result in CO_2_-related complications (22, 23). In recent years, the gasless TOETVA has been reported including ours (14), while TOaST without CO_2_ insufflation has not been studied. Nevertheless, gasless TOETVA still has unique drawbacks that are inherent to the approach because of the special anatomical structure of the oral vestibule. First, the mental nerves have a branch-like distribution on the right and left sides of the oral vestibule. The position of the midline intraoral incision and two lateral incisions described previously in TOETVA has a significant risk to lead to a high incidence of mental nerve injury with long-term sensory loss to the anterior chin (16). Second, in our experience, prolonged compression of the fixed chin by a 10-mm trocar in the observation port and the instrument leverage on the mandibular prominence possibly cause chin pain and discomfort in many patients. Furthermore, some patients report residual pain and tightness, which may be associated with the extent and range of the flap dissection (24). Third, creating a surgical space in TOETVA requires premandibular dissection and separation of the soft tissues from the bone, which is difficult and prone to the complication of skin perforation, especially in patients with prominent chin projection and submental depressions. Fourth, the circuitous route from the access point to the target area and the rather small space in the central region of the mandible may lead to an increased risk of capsular disruption during tumor extraction.

In this study, we develop the gasless STET with a novel suspension system intending to thoroughly address the risks associated with CO_2_ insufflation during the procedure and the series of drawbacks caused by inherent to the TOETVA while retaining the original benefits of TOETVA. We use the suspension retractor by attaching it to the anesthetic frame *via* a sterile bandage to eliminate complicated installation steps and reduce the cost of purchasing special equipment. In addition, it helps us prevent CO_2_-related complications, such as hypercapnia, tumor implantation, and even gas embolism. Smoke and water mist from energy instruments during operation could be continuously removed by a suction device on the suspension retractor to provide a clear surgical field of view for gasless STET. Less wiping of the endoscope lens also helps us reduce the procedure time. In summary, compared with the previous retraction technique involving percutaneously placed stitches in both TOETVA and TOaST (7, 25, 26), this novel suspension system allows for a more stable, sufficient and clear space for endoscopic thyroidectomy.

In addition, damage to the mental nerve is a TOETVA-unique complication, resulting in varying degrees of numbness around the chin. It has been demonstrated that the incidence of nerve injury can be effectively reduced by moving bilateral 5-mm incisions more lateral to the canines and closer to the free edge of the lower lip (17). Daqi et al. determined the location and distribution pattern of the mental nerve structures in relation to the oral vestibular incisions by cadaver dissection and showed that standard bilateral 5-mm incisions were safe for determining mental nerve integrity, while the transverse incision in the central oral vestibule was prone to injury of the branch of the mental nerve at a rate of 12–25% (18). Hence, the transverse incision of the oral vestibule is recommended to be moved to the submental area, away from the mental and other facial nerve branches, which theoretically reduces the probability of nerve injury. Additionally, by changing the position of the median transverse incision, long-term compression of the lower lip and chin nerve branches by the trocar can be avoided and the extent of flap dissection can be shortened, which is likely to further reduce postoperative discomfort. In this study, mild abnormal sensation of the lower lip occurred only in three patients (3/75, 4%) postoperatively, and all returned to normal one month later. This may have resulted from compression of the lip by trocars rather than injury to the mental nerve.

The process of surgical space establishment *via* the submental approach is not prone to the complication of skin perforation, even in patients with prominent chins and submental depressions. There were no cases of skin perforation in this study. Thanks to the change in the position of the main incision making it less difficult to establish the surgical space, our surgery time was even shorter at 141.1 ± 34.8 minutes for lobectomy + CND measured from the time of incision to the completion of skin suture. As the operators become more proficient at this procedure, the surgery time is expected to be further reduced.

Follicular lesions require evaluation of the intact tumor capsule to determine if there is evidence of invasion to diagnose malignancy, in which case tumor complete extraction is critical. When necessary, it is simpler to extend the submental incision laterally than using the transoral vestibular incision without concerns about damaging the mental nerves. Furthermore, the submental approach permits the extraction of larger specimens since it avoids the circuitous but required traversal path from the extraction site to the thyroid. As a result, the eligibility criteria for tumor size could be suitably adjusted. In this study, subject to ensuring complete excision and removal of the surgical specimen, the average size of benign tumors was 36.3 ± 11.2 mm, of which the largest tumor diameter was approximately 52.2 mm, and the average size of malignant tumors was 8.2 ± 4.9 mm, of which the largest tumor diameter was 3 cm.

In terms of cosmetic results, the submental incision was more discreet and less likely to cause scarring as the skin at the submental area was looser and located in the blind spot of the front view. No patient complained of not achieving the desired cosmetic results.

We found gasless STET is acceptably safe and feasible, meeting the criteria for open surgery and it provides bilateral access to the thyroid lobe as does TOETVA. Since we started gasless STET, there were 59 lobectomies with or without central neck dissection, and 16 total thyroidectomies with unilateral or bilateral central neck dissection, none of which were converted to conventional open surgery. All patients in our group had complete tumor resection and thorough lymph node dissection since the gasless STET permitted total-field exposure of the central region and posterior sternal lymph nodes. Our data presented a higher average number of totally resected lymph nodes, up to 7.2 ± 4.5 (27–29).

For surgical complications, the probability of occurrence of any complication was relatively low. As would be expected, there were no instances of permanent recurrent laryngeal nerve paralysis or hypoparathyroidism. Temporary hypoparathyroidism occurred in two patients, both of whom underwent total thyroidectomy and bilateral lymph node dissection in the central region, and both of whom had bilateral inferior parathyroid gland transplants intraoperatively. We recommend preserving the parathyroid glands *in situ* whenever possible, although these annoying symptoms tend to improve over time. One patient had a mild postoperative chylous fistula that improved after adjustment to a fat-free diet. Notably, gasless STET is no longer a traditional sterile incision; thus, it requires preoperative prophylactic use of antimicrobial medication and intraoperative disinfection, similar to the transoral vestibular approach. There was one case of subcutaneous redness and infection in this study, which was resolved with antimicrobial therapy. Additionally, during our average follow-up period of 13.6 ± 0.3 months, one patient was found to have a suspicious recurrence of lateral neck lymph nodes by ultrasound at the 6-month postoperative follow-up, and the postoperative pathology of this patient indicated a tumor size of 3 cm and 10 metastatic lymph nodes in the central compartment. Further studies on the efficacy of tumor treatment are needed because of the short follow-up period.

Our gasless STET still has several minor drawbacks. Specifically, gasless STET is somewhat restrictive in terms of who it is suitable for. In the premise of not having a scar constitution, patients with a prominent chin or those without a double chin are usually our preferred option for inclusion. If male patients have beards, then this procedure is more friendly to them. However, in others, especially patients with scarring, the incision in the submental area may not be completely hidden as in the TOETVA, thus affecting the aesthetics. Additionally, given the limited number of patients included in this study and the short follow-up period, a larger sample size and longer follow-up results are needed to further validate the importance of this surgical technique.

In conclusion, gasless STET using the innovative suspension system was shown to be a safe, feasible, and sensible alternative for selected patients. It has the potential to compensate for the shortcomings of mental nerve injury and gas embolism without sacrificing good cosmetic results to a certain extent, making it a new surgical approach for clinical application.

## Data availability statement

The raw data supporting the conclusions of this article will be made available by the authors, without undue reservation.

## Ethics statement

The studies involving human participants were reviewed and approved by Zhejiang University of Medicine School Sir Run Run Shaw Hospital. The patients/participants provided their written informed consent to participate in this study. Written informed consent was obtained from the individual(s) for the publication of any potentially identifiable images or data included in this article.

## Author contributions

DZ contributed to the conception and design of the study. LG and LX organized the database. GH, JC, JL, XL, and XJ performed the statistical analysis. JJ wrote the first draft of the manuscript. DZ and JJ contributed to the manuscript revision. All authors contributed to the article and approved the submitted version.
